# Atomic Configuration of Point Defect Clusters in Ion-Irradiated Silicon Carbide

**DOI:** 10.1038/s41598-017-15037-w

**Published:** 2017-11-07

**Authors:** Y. R. Lin, L. G. Chen, C. Y. Hsieh, M. T. Chang, K. Y. Fung, A. Hu, S. C. Lo, F. R. Chen, J. J. Kai

**Affiliations:** 1 National Tsing-Hua University, Department of Engineering and System Science, 30013 Hsinchu, Taiwan; 2 Industrial Technology Research Institute, Material and Chemical Research Laboratories, 31040 Hsinchu, Taiwan; 3 The City University of Hong Kong, Department of Mechanical and Biomedical Engineering, 852 Kowloon, Kowloon, Hong Kong

## Abstract

Silicon Carbide (SiC) is a promising cladding material for accident-tolerant fuel in light water reactors due to its excellent resistance to chemical attacks at high temperatures, which can prevent severe accident-induced environmental disasters. Although it has been known for decades that radiation-induced swelling at low temperatures is driven by the formation of black spot defects with sizes smaller than 2 nm in irradiated SiC, the structure of these defect clusters and the mechanism of lattice expansion have not been clarified and remain as one of the most important scientific issues in nuclear materials research. Here we report the atomic configuration of defect clusters using Cs-corrected transmission electron microscopy and molecular dynamics to determine the mechanism of these defects to radiation swelling. This study also provides compelling evidence that irradiation-induced point defect clusters are vacancy-rich clusters and lattice expansion results from the homogenous distribution of unrecovered interstitials in the material.

## Introduction

Silicon carbide (SiC) is a wide-band gap semiconductor^1^, a key refractory ceramic^2^, and a radiation-tolerant structural material^3^ that can be functionalized by ion-implantation doping^4,5^ and has great potential for device and structural applications in the electronic industry and nuclear radiation environments. With the development of semiconductors and the mission to improve the safety of nuclear reactors, SiC has increased in attractiveness. Irradiation-induced defects in SiC at temperatures above 1000 °C have been observed and analyzed in detail^6–8^. However, in a previous study^3^, the observable microstructure of neutrons and ion irradiated SiC below 1000 °C was described as containing black spot defects (BSDs), mostly circular or oval in shape, which appeared as nanometer scale black spots in bright field transmission electron microscopy (TEM) images. While BSDs are believed to associate with radiation-induced swelling, the detailed internal structure of a BSD is unknown. If left unchecked, the swelling arising from these undefined defects may lead to unwanted degradation of the mechanical properties of SiC. Besides nuclear applications, Cubic SiC (3C-SiC) exhibits excellent electrical properties, which have great potential for electronic devices. However, SiC-based electronic devices have not achieved their expected performance thus far, mainly due to defects formed during the manufacturing process^9^. Therefore, in order to use SiC or functionalize it by ion-implantation doping in electronic devices, a fundamental understanding of the microstructural evolution of this material is also crucial.

BSDs, types of point defect cluster composed by vacancies and interstitials in irradiated SiC, have been characterized mainly using TEM by many researchers. However, there is little understanding of the structure of these nano-scale clusters since imaging an inhomogeneous distribution of atoms buried inside a host material is required. High-angle annular dark-field scanning transmission electron microscopy (HAADF-STEM) is an imaging technique which can detect individual atoms at an atomic resolution^10^. The annular-shaped high-angle detector behind the sample collects the signal dominated by Rutherford and thermal diffuse scattering. When applied in a restricted zone-axis orientation, the HAADF scattering signal from a single column of atoms is strongly dependent on the atomic number (roughly Z^1.7^, hence it is also referred to as Z-contrast images) and the thickness of the sample^11,12^. On the other hand, the annular bright-field (ABF) imaging technique collecting lower-angle signals is able to directly detect the position of light atoms^13,14^ (e.g., oxygen, lithium, and carbon, which cannot be significantly imaged by HAADF images). In this study, we systematically characterized the atomic structure of the nanoclusters in irradiated 3C-SiC using a Cs-corrected STEM (JEOL, JEM-ARM200F) at an accelerating voltage of 200 kV with an ultra-high spatial resolution of approximately 0.12 nm.

## Results

**Figure 1:**
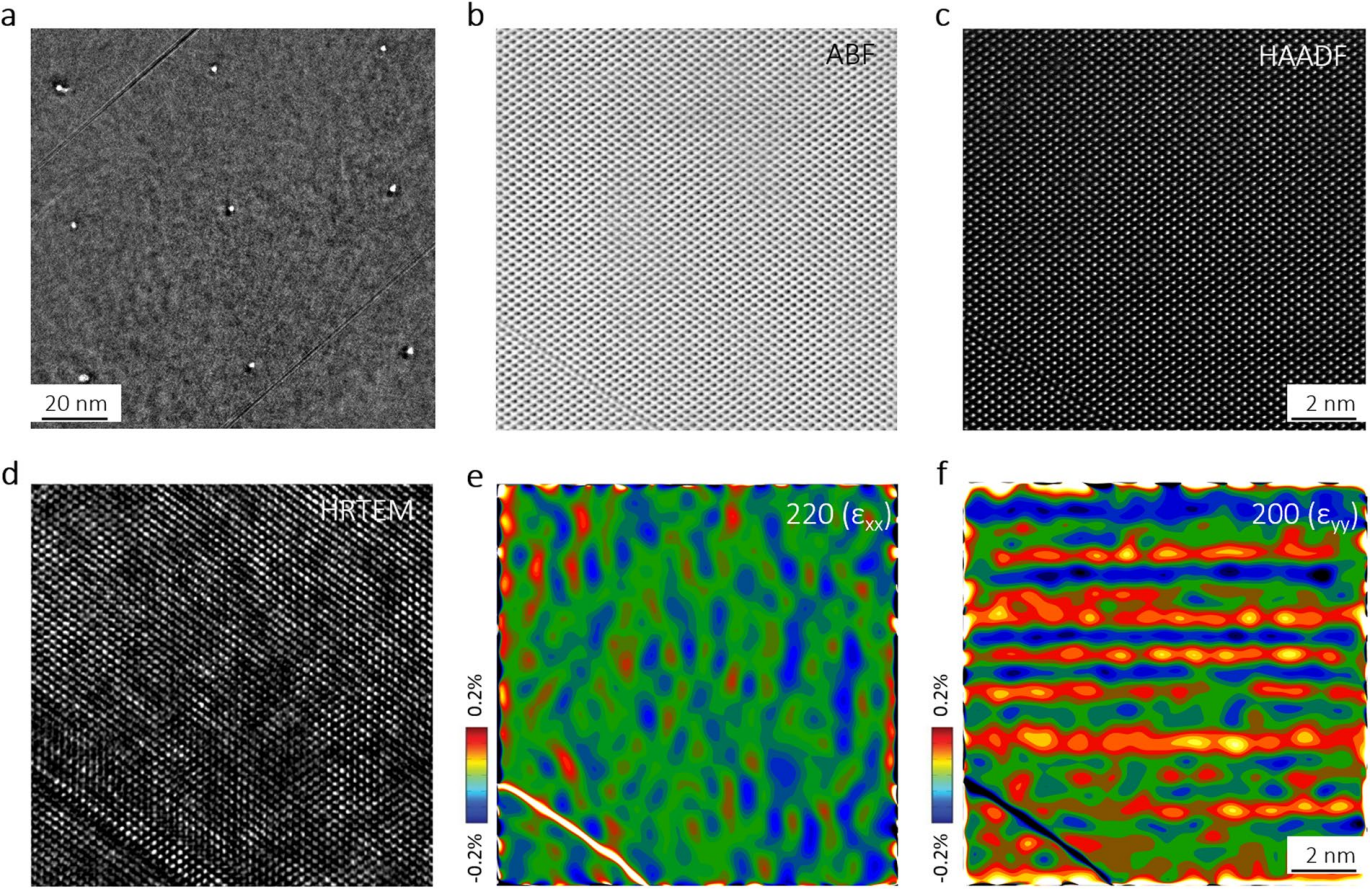
TEM and STEM analysis of 20 dpa ion irradiated 3C-SiC. (**a**) Nine artificially created holes to locate and identify a single black spot defect shown in a TEM image; (**b**) Experimental ABF-TEM; (**c**) HAADF-STEM; and (**d**) HR-TEM image in the [011] zone-axis orientation at the same sample regime; and (**e**) Experimental strain components ε_xx_ and (**f**) ε_yy_ derived from b by geometric phase analysis.

We discovered the regions including these black spot defects under TEM images for SiC samples irradiated by 5.1 MeV Si-ions under 400 °C and 20 dPa. Focusing the electron beam of the TEM, we artificially constructed nine holes to locate and identify a region including a black dot structure (Fig. 1a) in order to exactly observe the same region of atom columns when shifting the microscope into the ABF-STEM (Fig. 1b), HAADF-STEM (Fig. 1c), and HR-TEM (Fig. 1d) modes. The lower left corner of Fig. 1b,c and d indicates a primitive stacking fault which was mainly formed during the Chemical Vapor Deposition (CVD) lattice growing process due to a large lattice mismatch between SiC and Si substrate^8^. This primitive linear defect also allowed us to further align the atom columns in ABF-STEM, HAADF-STEM, and HR-TEM images one-to-one. In Fig. 1d, the image contrast and distortion between the region containing BSDs and the region of orderly arranged atoms are evidently higher than the ABF-STEM (Fig. 1b) and HAADF-STEM (Fig. 1c) images. It is important to understand that the higher intensity or deformity of the bright dots cannot precisely indicate the presence of an atom column in the HR-TEM image. Therefore, black spots observed under traditional TEM or HR-TEM are not always “defects”, but contrast caused by strain near the real “defects” in the material. On the other hand, HAADF-STEM imaging was used for structural characterization. This technique, which uses incoherent electrons, avoids the phase contrast effect and allows direct imaging of atomic locations, thereby overcoming the uncertainties caused by dynamical diffraction, delocalization, and interference in HR-TEM imaging.

Since image contrast and distortion in HR-TEM images may arise from the lattice strains caused by defect atoms^15^, we extracted strain information from the Z-contrast HAADF-STEM images (Fig. 1c) based on a geometrical phase analysis (GPA)^16^. Fig. 1e shows the strain mapping result along <220>, where the blue regions with relatively negative strains match the dark contrast region in HR-TEM images (Fig. 1d). The strain mapping result along <002> (Fig. 1f) shows that the strain was highly orientated along the <002> direction. This result matches our previously Synchrotron-based XRD analysis of the surface (002)^8^, which has the same orientation as the 3C-SiC crystal deposited on the Si substrate, and shows the largest expansion value suggested to be caused by <100> dumbbells^17^. In summary, strain mapping along the <220> direction may not be affected by the long ranged <100> direction strains caused by dumbbells, which distributed homogeneously in the material, but reveals the locally short range strains caused by point defect clusters. Considering the diffusivity of point defects, it is generally accepted that vacancies may be frozen due to low diffusion when the temperature is below 1/3 T_m_ (materials melting point). These defects cannot migrate to their sinks or evolve to other microstructures. For 3C-SiC, according to the previous reports^18,19^, the diffusion length can be calculated by the Arrhenius’ equation. This equation shows that, in 400 °C, both Si and C vacancies are immobile and the diffusion coefficient of C (~9.24 × 10^−9^ cm^2^/s) is over six orders of magnitude than that of Si (~4.07 × 10^−15^ cm^2^/s). However, Si and C interstitials with a high rate of self-diffusion are reasonable to move and compose dumbbells which show low formation energies in former molecular dynamic (MD) studies^20,21^. Thus, unrecovered vacancies may form during the process of displacement spike and remain immobile in the black spot damaged region (observed under traditional TEM). As for interstitials, separated in the material, they may remain as single point defects or become dumbbells, since the amounts of interstitials and vacancies should be equal when producing Frankel pairs.

**Figure 2:**
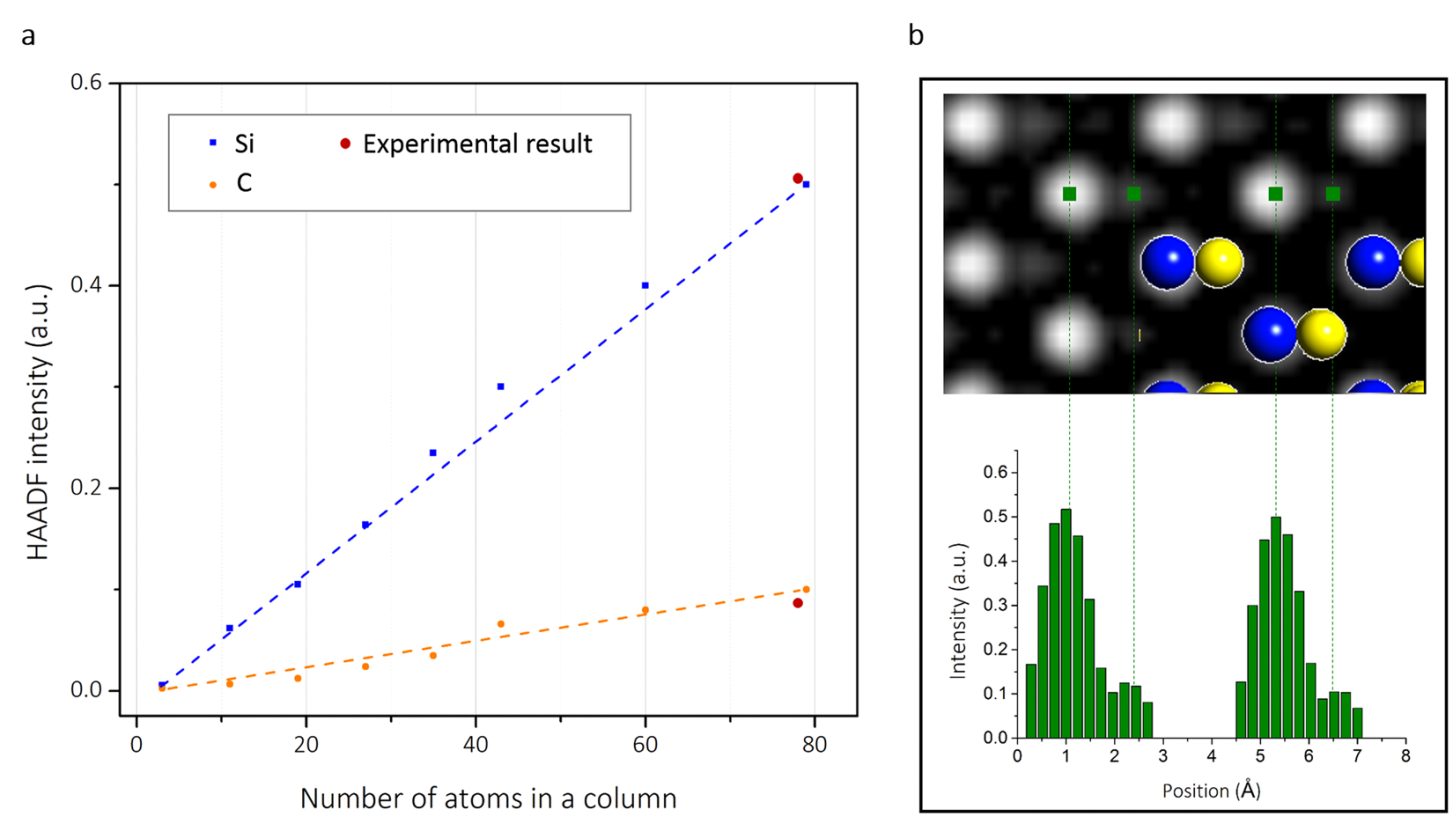
Quantification of HAADF-STEM images of SiC. (**a**) Linear increase of the estimated mean intensity values with an increasing number of Si and C atoms in a column oriented along the [011] direction. (**b**) Simulated HAADF images and the intensity structure model for the Si column (blue) and the C column (yellow) with 79 atoms oriented along the [011] direction.

**Figure 3:**
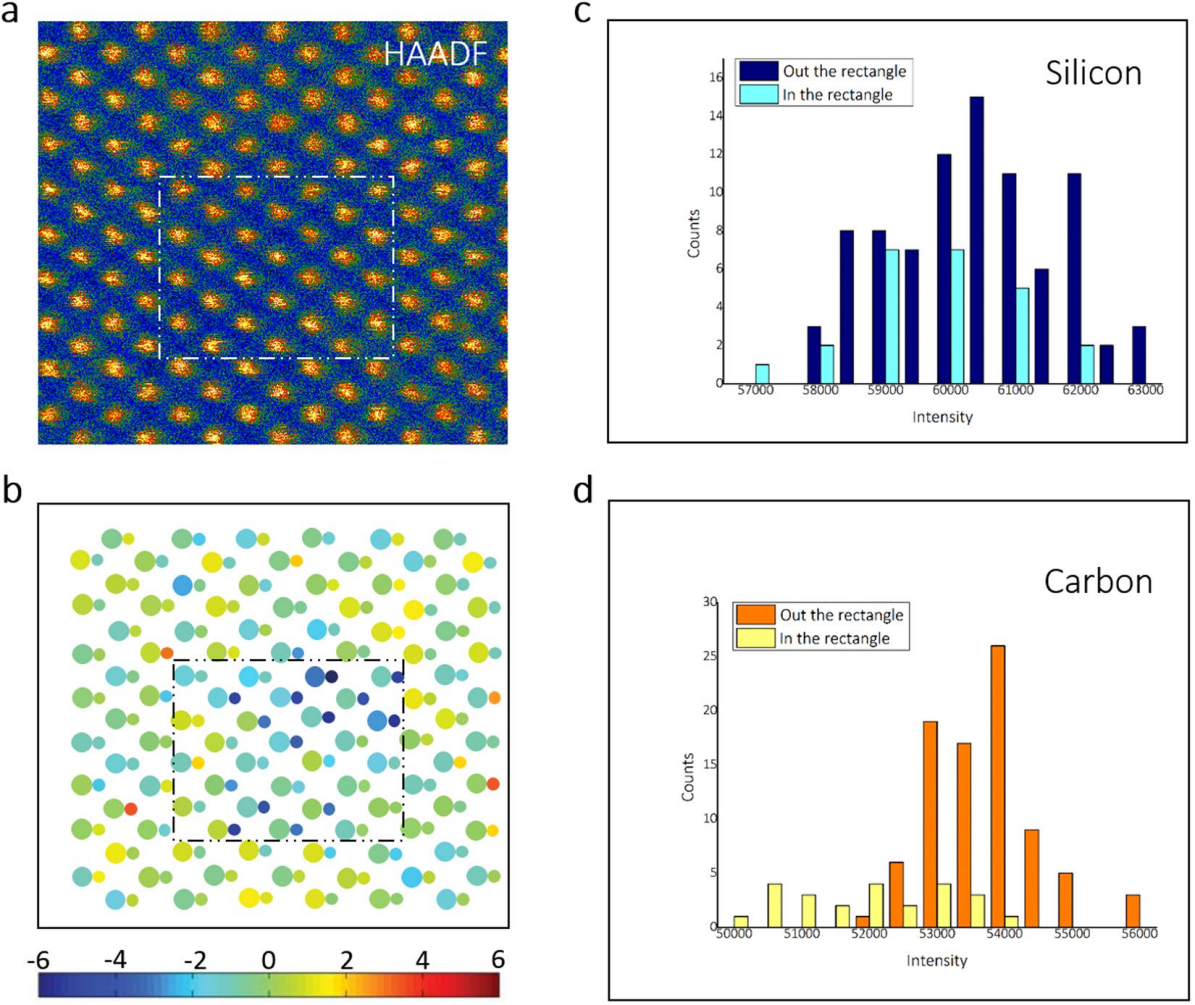
Quantification of HAADF-STEM images of a point defect cluster. (**a**) HAADF-STEM image of Fig. 3a shown in pseudo-color; (**b**) Difference between the computed atom counts by the experiment in the [011] zone-axis orientation; and (**c**) Histogram of scattered intensities inside and outside of the box of Silicon and (**d**) Carbon columns.

To confirm this concept, a detailed element distribution in the region containing BSDs was investigated through a quantitative method to analyze HAADF-STEM images^11,22^, in which atomic columns with a difference in (average) atomic number could be identified and the scattered intensities could be computed (see the Supplementary Information). These intensities scale with the average atomic number Z, providing the ability to distinguish columns containing a certain amount of vacancies or interstitials from pure columns (Fig. 2b). The average intensity of atom columns in HAADF experimental images with sample thickness ~25 nm (shown as red spots in Fig. 2a.) agrees with the simulated intensity of the crystal modal built by CrystalKit software. In detail, intensity at each atomic-column was averaged within a circular mask and compared with simulated values. Further, the thickness of the sample was estimated by using an intensity of zero loss and plasma loss in EELS spectra. Comparison of the intensity of the Si and C atom columns inside and outside the box shows that the intensity of Si (Fig. 3c) has no significant difference while that of C (Fig. 3d) has a lower average inside the box. Refined models describing the contrast of the atom columns enclosed in the white box of Fig. 3a are presented in Fig. 3b. In this method, 28 atom columns inside the box averagely lost 0.94 and 2.99 atoms per column for Si and C, respectively. This result is also in agreement with the high diffusivity of carbon interstitials as mentioned earlier. In addition, around the box, some columns contained excess atoms (Fig. 3b), which were brighter in the HAADF-STEM images (Fig. 3a). These signals may originate from the interstitial defects.

**Figure 4:**
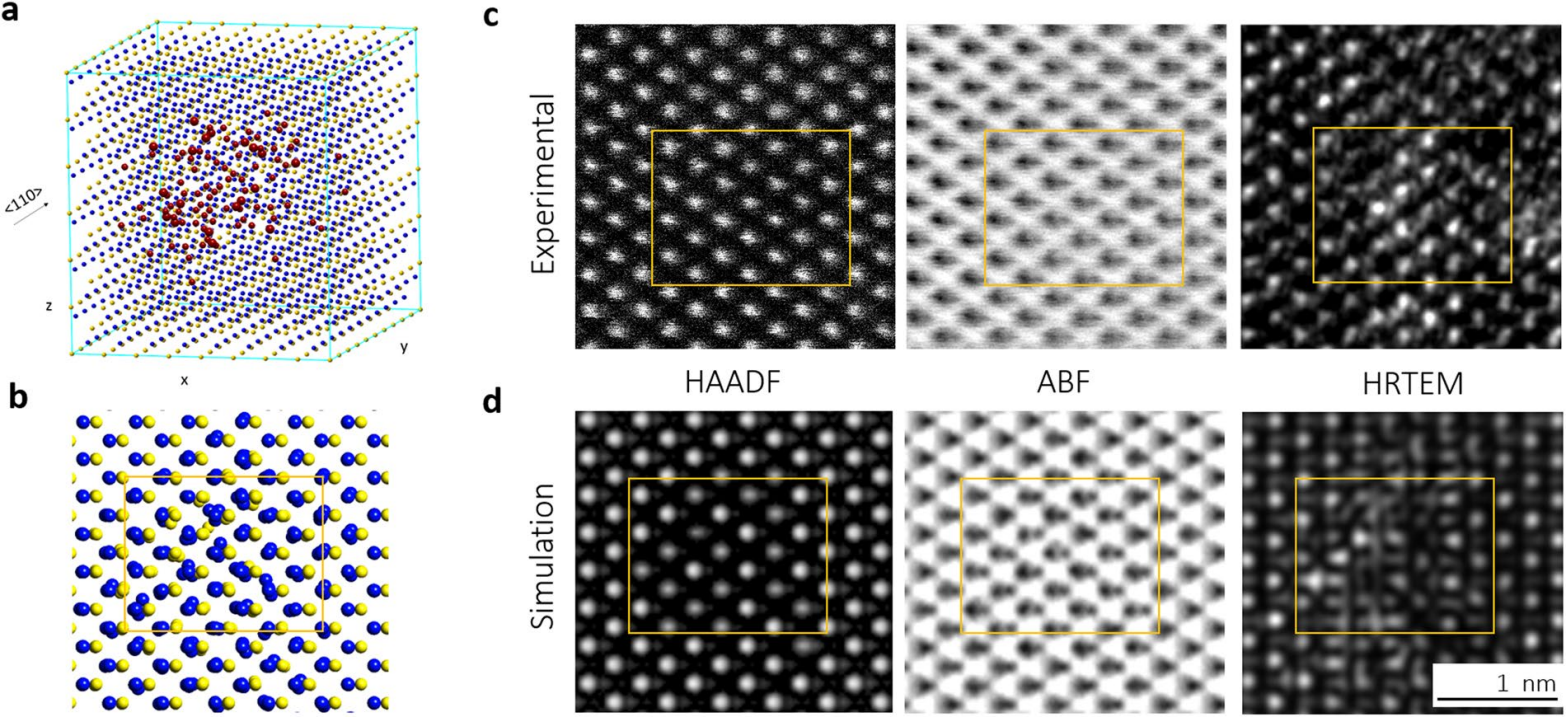
Experimental and Simulated TEM images of a point defect cluster. (**a**) Structure model with removed atoms (red) based on Fig. 3b; (**b**) Structure model of Fig. 4a relaxed through the MD method (Si in blue and C in yellow); and (**c**) Experimental and (**d**) Simulated images of HAADF-STEM, ABF-STEM, and HR-TEM characterization of the point defect cluster. The same point defect cluster in the box areas was characterized in different image modes in the [011] zone-axis orientation.

To distinguish the difference between these STEM and HRTEM images, image simulation was carried out for the experimental results. From the results of the atom count, approximately 64% of atoms estimated were absent (26 for Si and 83 for C.) in a sphere with a diameter of approximately 1.5 nm containing 168 atoms. To understand the atomic structure of the point defect cluster, we constructed a possible structural model in which a point defect cluster is embedded in the 3C-SiC matrix. Vacancy atoms were positioned at the center of a diamond cubic crystal structure matrix box with dimensions of 3.08 nm × 2.61 nm × 2.46 nm which included 1,823 atoms (Fig. 4a). Firstly, we introduced the same number of vacancies and interstitials obtained through the atom counting result in the model, and then relaxed the structure to the lowest energy state using an MD method^16,23^. During the relaxation, the neighboring atoms of vacancies moved towards the open space, leading to a certain lattice distortion. Secondly, the final structural models of the atomic arrangement were built by CrystalKitX software, (Fig. 4b) and image simulations were carried out with the help of MacTempasX software. The images simulated under identical conditions (Fig. 4d) agreed well with the experimental images (Fig. 4c). In both simulated and experimental images, our investigation using HAADF-STEM shows that vacancy-rich cluster were observed in the same region containing black spot defects observed in HR images, but the position of the “black dots” dose not exactly match. For example, in Fig. 1, BSD’s in the HRTEM image (Fig. 1d) shows dark contrast but the HAADF image (Fig. 1c) shows bright contrast. This discrepancy is due to the difference between the imaging principle of HRTEM (images with phase contrast) and HAADF images.

Further, in order to make sure the consistency between theoretical calculation and experimental result, a series of 3C-SiC ion-irradiated cascade MD simulations was carried out by LAMMPS^24^. The ensemble simulation results also show that the defect clusters produced by ion-irradiation of 3C-SiC are generally vacancy-rich, and the number of isolated interstitial are much larger than that of isolated vacancy.

## Discussion

Previously, BSDs were suggested to be tiny clusters of self-interstitial atoms in various indeterminate configurations^3,18,19^. When the temperature is above 600 °C, small {111} interstitial loops were observed with surrounding black contrast regions when the zone-axis was fixed along [011]^6,8^. Further, researchers have suggested that vacancies remain as a single point defect or a di-vacancy defect inside the irradiated samples that are too small to be observed under TEM^25^. However, the point defect concentration calculated from these defects under TEM images indicates that the concentration of total vacancies was still far less compared to that of the interstitials^7^. According to the results of this study, vacancy-rich areas at a scale of approximately 2 nm were observed under HAADF images. Comparing HAADF images with HRTEM images (Fig. 4c and d), the atom columns without atoms had a darker contrast in the HAADF images, but were brighter in the HRTEM images. This brightness was likely due to the mass-thickness contrast in the HRTEM images. On the other hand, the darker region was also observed in both the experimental and simulated images nearby these bright spots in the HRTEM images (Fig. 4c and d). These spots likely arise from phase contrast in the HRTEM images, which mainly results from lattice strain as shown in Fig. 1e. Nevertheless, although the ABF images provide partial phase contrast images, they show a lighter contrast which the dark spots of Si and C atom column seems more separated in the atom columns absent of atoms. These ABF images also support the result of the HAADF images. In other words, BSDs observed under traditional TEM may not exactly indicate the position and the number of atoms along a column of atoms. Dark contrast shown in the HRTEM could mainly contributed by the strain near a point defect cluster, which even has a brighter contrast under HR images. We propose that most of the point defect clusters observed by STEM in this irradiation condition should be vacancy-rich clusters, where interstitials appear around a vacancy-rich core (Fig. 3b). In addition, these knock-out mobile interstitials may diffuse and connect with other atoms to constitute new structures such as dumbbells and loops. In fact, this vacancy cluster structure as black dots in the electron micrograph was first predicted in 1956 by the radiation-damage theory and is known as the depleted zone or a displacement spike^26^.

Our observations demonstrate the structure and properties of point defect clusters in irradiated SiC at 400 °C. Further, the BSDs observed under traditional TEM may not exactly indicate the position and the number of atoms of a point defect cluster. In short, point defect clusters identified in this study were carbon vacancy-rich depleted zones. Understanding the structure of these defects that were once unable to be visualized in SiC has significant implications for the study of irradiation effects in other ceramics for applications in extreme radiation environments. Scientific advances based on this work will not only facilitate the design of radiation resistant or low defect density materials for advanced nuclear power plants and the electronics industry, but will also contribute as a foundation for the development and control of desirable material properties. This foundation will enable broad advances in environmental security, sustainable energy technologies, and device fabrication involving materials subjected to severe radiation environments or ion beam modification.

## Methods

### Ion irradiation

A 3C-SiC crystal with surface orientation (002), grown using the CVD process on a Si substrate (by NOVA SiC, France) was irradiated at the DuET facility at Kyoto University, Japan. A 5.1 MeV Si^2+^ ion with a fluence of 5.65 × 10^17^ ion/cm^2^ was implanted for inducing displacement damage at irradiation temperatures of 400 °C.

### TEM characterization

Samples were prepared for TEM and STEM analysis using a focused ion beam (FIB) in a cross beam Zeiss Auriga FIB/SEM. Irradiated 3C-SiC was prepared using a gallium FIB beam probe at 1 KeV to minimize FIB beam damage. The thickness of the sample was estimated by using the intensity of zero loss and plasma loss in EELS spectra for approximately 25 nm^27^. STEM imaging was performed on various samples in a Cs-corrected STEM (JEOL, JEM-ARM200F) at an accelerating voltage of 200 kV. The probe convergence angle was 27 mrad. The HAADF detector was set to collect electrons scattered between 60 mrad and 160 mrad, and the ABF detector was set to collect electrons scattered between 11 mrad and 23 mrad.

### Structure modeling and image simulation

The structure models for image simulation were constructed by LAMMPS molecular dynamics simulation. First, a cubic SiC structure including a vacancy-rich structure as an initial structure was prepared. The structure was then annealed at 400 °C for structure relaxation based on the Gao-Weber/ZBL potential^28^. The annealing time was 2 ps, and a further increase in time did not cause any obvious structural change. Structural models of the atomic arrangement were built by the CrystalKitX software, and STEM image simulation was carried out with the help of the MacTempasX software. In the calculations, the results were obtained at a zero defocus and a 27 mrad probe semi-angle for both simulations corresponding to a probe size of 0.12 nm. Detailed information of the probe convergence angle and HAADF and ABF detector inner and outer angles are shown in the Supplementary Table 1.

## Electronic supplementary material

Supplementary Information
