# Supplementary material for: Atomic Configuration of Point Defect Clusters in Ion-Irradiated Silicon Carbide
Source: Sci Rep. 2017 Nov 7;7:14635. doi: 10.1038/s41598-017-15037-w (PMC5676775; doi:10.1038/s41598-017-15037-w)
Supplement: Supplementary file 1 — Supplementary Information [file 41598_2017_15037_MOESM1_ESM.pdf]

Atomic Configuration of Point Defect Clusters in Ion-Irradiated Silicon Car-bide

Y.R. Lin, L.G. Chen, C.Y. Hsieh, M.T. Chang, K.Y. Fung, A. Hu, S.C. Lo, F.R. Chen and J.J. Kai

*Corresponding author: ceer0001@gmail.com, jjkai34280@gmail.com

Supplementary Information

Ion irradiation

The materials used in this study were a single crystal 3C-SiC wafer (CVD process by NOVA SiC, France) and a 3C-SiC crystal with surface orientation (002) deposited on a Si substrate. The thickness of the SiC epitaxy layer and Si substrate were 1.17 μm and 450 μm, respectively. In Supplementary Fig. 1, the depth distribution versus displacement per atom of incident Si ion was simulated by SRIM code simulation, and the displacement energies for Si and C were set at 35 eV and 21 eV, respectively. From examination of the microstructure, the displacement damage level, calculated at a depth of 0.6 μm from the irradiated surface, was 20 dpa and the damage rate was about 2.7 x 10^{-3} dpa/s averaged over the damage range. In order to avoid the accumulation of the implanted Si ion in the SiC layer, specific thickness of the epitaxy layer and implanted ion energy were chosen.

Quantification of local information from HAADF images

We employed a recently developed method that allowed the measurement of the total intensity of the scattered electrons for each atomic column.[1, 2] The HAADF simulation was carried out with the commercial software MacTempas, which utilizes a frozen phonon model. An overview of parameters set for the image simulation is shown in Supplementary Table 1. Here we set up different structural models of different thicknesses (numbers of atoms) of Si and C columns. During the image simulating process, these structures were viewed in the zone-axis of [011] orientation as same as the experimental images, and the intensity of each Si and C atom column was recorded. This is shown more clearly in Fig. 2 in the main text where the normalized intensity values are shown as a function of the number of Si and C atoms in a column together with a straight line through these values using linear regression. The

equation used to calculate the ratio of the intensities of the elements or compounds is

$$\mathrm{I} = \mathrm{I}_{bg} + B\times\{[\rho/A\times n)\times t\times Z^{\alpha}]\} \quad (1)$$

where I_{bg} denotes the intensity of the background; ρ represents the density of the material; A indicates the atomic weight; n represents the neutron mass; t stands for the thickness of the sample; B and α denote parameters; and Z represents the atomic number. In order to extract quantitative information from the HAADF STEM image, certain parameters have to be kept constant for calibration purposes. Fig. 3b shows the corresponding atomic number map after a quantitative comparison between the simulated and experimental HAADF intensities and a quantitative analysis of the intensity histogram. In addition, the Z-power law dependence of the intensity for the two types of columns identified in the sample on their atomic number Z can be written as

$$\frac{I_{si}}{I_C} \propto (\frac{Z_{si}}{Z_C})^{\alpha} \quad (2)$$

According to this equation, the Z dependent power law measured for this case is 1.88 at a thickness of 25 nm (~80 atoms).

Geometrical phase analysis

Previous research suggests that a properly chosen Fourier mask size and strain profile direction/width enables an accuracy of 1 pm for GPA strain quantification in the STEM fast-scan direction with a spatial resolution of <1 nm.[3] In this work, a GPA program called FRERtools, a Digital Micrograph plug-in developed by C.T. Koch et al., was used for the GPA.[4] The geometric phase algorithm reconstructs the displacement field utilizing Fourier filtering centered around two non-collinear Bragg vectors of the power spectrum generated from a high-resolution micrograph. The vectorial representation of the displacement field is:

$$\mathrm{u}(r) = \left(\frac{-1}{2}\pi\right)[Pg_1(r)\times a_1 + Pg_2(r)\times a_2] \quad (3)$$

where r represents a position in the image; a_1 and a_2 denote the associated lattice vectors of g_1 and g_2 in real space; and $Pg_1(r)$ and $Pg_2(r)$ are two geometric phase images representing the local deviation in two sets of lattice planes selected from a reference lattice. The phase in the GPA does not refer to the electron wave function, but to the position of image contrast maxima; hence, it is referred to as the geometric phase. The HAADF-STEM image used in this study with its Fast Fourier Transformation result is shown in Supplementary Fig. 2. For the cubic structures, two low-order vertical vectors g_1 = [220] and g_2 = [002] were selected. Then, the derivation of the displacement field gives the strain field in a principal direction as

$$\varepsilon_{xx} = \frac{\partial \mathrm{u}_x(r)}{\partial x} \quad (4)$$

$$\varepsilon_{yy} = \frac{\partial \mathrm{u}_y(r)}{\partial y} \quad (5)$$

Thus, the biaxial strains ε_{xx} and ε_{yy} are derived to illustrate the local lattice displacement from the reference lattice.[5, 6]

Note that the strain determined by the GPA here is a relative value. For example, in the case of interface lattice strain, the substrate lattices are usually selected as a reference. The sign of ε indicates whether the measured local lattice is larger (+) or smaller (−) than the reference lattice. The relative strain can be easily transformed into the conventional strain when the reference lattice parameter and the bulk material lattice are known. In our previous study, the linear expansion characterized by synchrotron-based XRD of [002] and [220] were 0.96% and 0.25%, respectively. The GPA generates strain maps with color contours directly illustrating the location of the relative strain. In this paper, a scale range of −0.2% to +0.2% was applied to all strain maps for consistency.

Supplementary Table. 1 | Overview of parameters set for the image simulation.

Voltage	200 KeV
Probe size	1.2 Å
Probe Semi-Angle	27 mrad
Inner Aperture	40 mrad
Outer Aperture	160 mrad

Figure Legends:

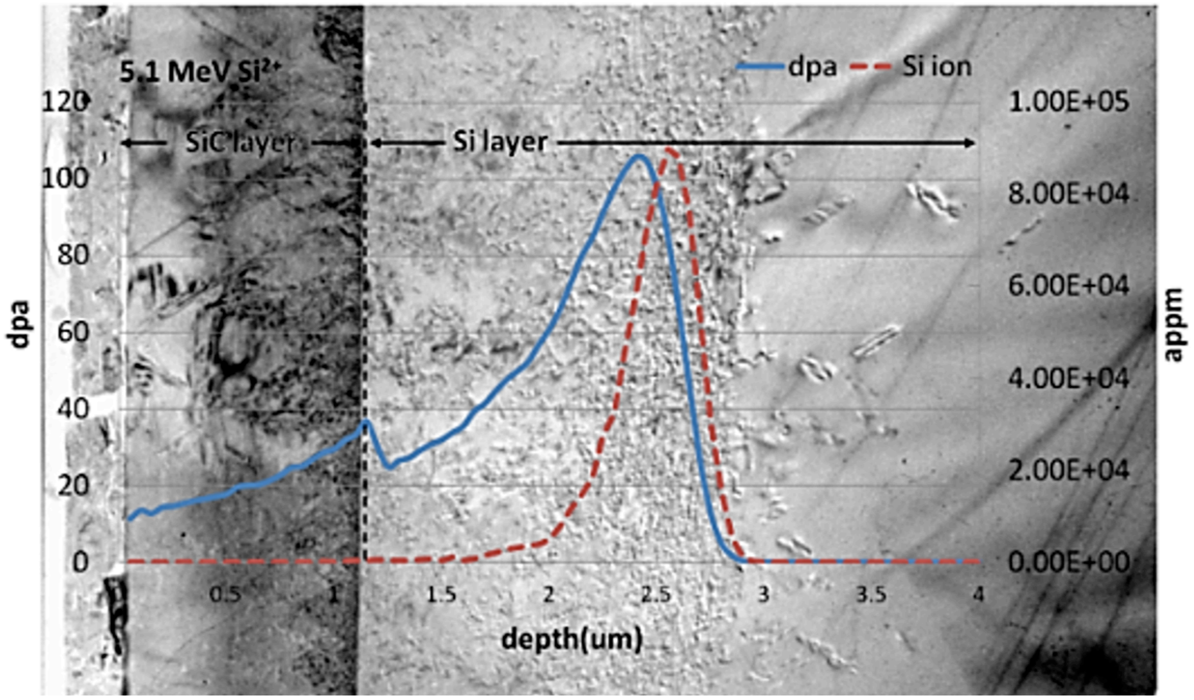

Supplementary Fig. 1 | Depth profile of the atomic displacement damage profile from a TEM cross-sectional image.

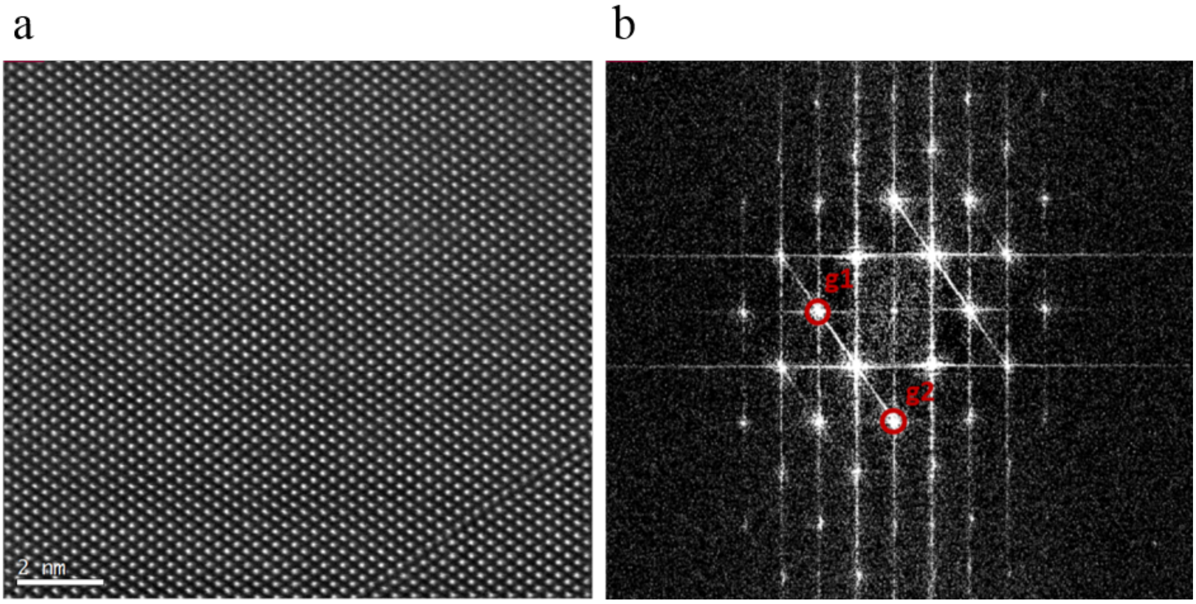

Supplementary Fig. 2 | Fast Fourier transform of a HAADF image of irradiated SiC. a: The HAADF STEM image oriented along [011] and **b:** The Fast Fourier transform of Fig. a.

Supplementary References

1. Jones, L. *et al.* Quantitative ADF STEM: acquisition, analysis and interpretation. *Mater. Sci. Eng*. **109**, 012008 (2016).
2. Chen, F.-R., Van Dyck, D. & Kisielowski, C. In-line three-dimensional holography of nanocrystalline objects at atomic resolution. *Nat. Commun*. **7**, 10603 (2016).
3. Zhu, Y, *et al.* Interface lattice displacement measurement to 1 pm by geometricphase analysis on aberration-corrected HAADF STEM images, *Acta Materialia* **61**, 5646–5663(2013)
4. Koch, C.T., Özdöl, V.B. and van Aken, P.A., An efficient, simple, and precise way to map strain with nanometer resolution in semiconductor devices, Applied Physics Letters 96 091901(2010)
5. Hytch, M.J., Snoeck, E., and Kilaas, R., Quantitative measurement of displacement and

strain fields from HREM micrographs. *Ultramicroscopy* **74**, 131 (1998).

6. Rouviere, J.L., and Sarigiannidou, E., Theoretical discussions on the geometrical phase analysis. *Ultramicroscopy* **106**, 1 (2005).
